# Clinical characteristics of brain metastases from lung cancer according to histological type: Pretreatment evaluation and survival following whole-brain radiotherapy

**DOI:** 10.3892/mco.2013.130

**Published:** 2013-05-23

**Authors:** TETSUYA KOMATSU, ETSUO KUNIEDA, YUKIO OIZUMI, YOSHIFUMI TAMAI, TAKESHI AKIBA

**Affiliations:** Department of Radiation Oncology, Tokai University School of Medicine, Isehara, Kanagawa 259-1193, Japan

**Keywords:** tyrosine kinase inhibitors, peritumoral edema

## Abstract

The histological type of lung cancer in patients with brain metastases may affect response to treatment and survival. We evaluated the clinical characteristics of brain metastases from lung cancer according to histological type in 70 consecutive patients with brain metastases from histologically confirmed lung cancer, who had been previously treated with whole-brain radiotherapy (WBRT). Histological type was divided into three categories: adenocarcinoma, small-cell lung carcinoma (SCLC) and other non-small cell lung cancer (NSCLC). The number, size and maximum diameter of brain metastases, the size and maximum diameter of peritumoral edema, the ratio of tumor and peritumoral edema, the asymptomatic ratio, the tumor size reduction rate, the complete response (CR) rate, the intracranial progression-free survival (PFS) and the overall survival (OS) were also evaluated. The median survival time for all patients was 26.2 weeks. Patients with SCLC exhibited a significantly smaller edema size and maximum diameter of edema compared to patients with other NSCLC (P=0.016 and 0.010, respectively). The ratio of tumor and peritumoral edema was also significantly lower in patients with SCLC compared to that in patients with adenocarcinoma and other NSCLC (P= 0.001). Significant differences in intracranial PFS and OS between adenocarcinoma and other NSCLC were also observed (P=0.018 and 0.004, respectively). Patients with adenocarcinoma who were treated with epidermal growth factor receptor (EGFR) tyrosine kinase inhibitors (TKIs) following WBRT, demonstrated a significant improvement in intracranial PFS and OS (P=0.008 and 0.004, respectively). The findings presented in this study may provide useful information for the management of brain metastases. Patients with SCLC exhibit a tendency to develop peritumoral edema to a lesser extent, compared to patients with other histological tumor types. Findings in the present study suggest that patients with adenocarcinoma, particularly those treated with EGFR-TKIs, exhibit improved survival rates.

## Introduction

Many cancer patients develop brain metastases, which lead to significant morbidity and decreased survival. Although no significant difference in survival has been reported in patients with different primary lesions (1), a previous large retrospective study revealed that the prognosis following the development of brain metastases was associated with the type of the primary lesion (2). The American Society for Radiation Oncology guidelines recommended that the results of randomized trials be interpreted with caution, due to the inclusion of patients with different primary cancers (3). However, lung cancer is the most common primary tumor accounting for more than half of all cases of brain metastases (2,4). Approximately 25% of patients with lung cancer develop brain metastases during the course of their illness (5,6) and their prognosis is poor, with a median survival time (MST) of 3–5 months (4–6).

Treatment approaches for patients with brain metastases vary depending on the lesion number and size. Selected patients with a potentially favorable prognosis may be suitable for stereotactic radiosurgery (SRS) (3). However, for patients with a large tumor or those with >3 lesions, whole-brain radiotherapy (WBRT) alone remains the standard treatment. The histological type of lung cancer may affect response to treatment and survival. Newly developed drugs and epidermal growth factor receptor (EGFR) tyrosine kinase inhibitors (TKIs) have been shown to affect treatment outcome, depending on the histological tumor type (7–9). These findings suggested that the histological assessment of lesions in patients with brain metastases from lung cancer may be beneficial. However, the effect of histological type on survival remains controversial. Bergqvist *et al* noted that adenocarcinoma was associated with the most favorable prognosis (10). Similarly, Kepka *et al* reported that patients with adenocarcinoma had a better prognosis compared to those with squamous cell carcinoma (11). Conversely, other studies reported that the survival time following WBRT did not differ among different histological types (12–14).

Although the effect of histological type on survival following WBRT remains unclear, previous studies have indicated that the use of EGFR-TKIs for the treatment of brain metastases following WBRT may affect patient survival (15). The aim of this study was to assess the survival of patients with lung cancer, defined according to histological type, following WBRT. In addition, pretreatment clinical characteristics, particularly peritumoral edema, were evaluated according to histological type, since few studies have addressed this subject. This retrospective study assessed the pre- and post-treatment features of brain metastases from lung cancer according to histological type and may provide beneficial information for the management of brain metastases in lung cancer patients.

## Patients and methods

### Patients and classification

The present study was approved by the Institutional Review Board of our hospital (12R072). Between September, 2005 and April, 2011, a total of 102 patients underwent WBRT alone, excluding prophylactic cranial irradiation, for brain metastases from lung cancer at our institution. No patients underwent planned WBRT in combination with surgery or stereotactic radiosurgery (SRS). Out of the 102 patients, 94 patients completed the prescribed radiation dose. To evaluate the pretreatment characteristics by imaging, selection was limited to patients who underwent contrast-enhanced magnetic resonance imaging (MRI) within 2 weeks prior to the initiation of radiotherapy. Thirteen patients who had been diagnosed using computed tomography or non-contrast-enhanced MRI were excluded. Nine patients in whom the histological type of the primary tumor could not be determined and 2 patients with carcinomatous meningitis were also excluded. The remaining 70 patients underwent WBRT within 2 weeks after the diagnosis of brain metastases. WBRT was performed using lateral opposed fields with a 6-MV linear accelerator. Lesions were divided into 3 categories according to histopathological type: adenocarcinoma, small-cell lung carcinoma (SCLC) and other non-small cell lung cancer (NSCLC). Recursive partitioning analysis (RPA) as suggested by Gaspar *et al* (16) was evaluated in all 70 patients.

### Assessment of pretreatment factors

To determine pretreatment clinical characteristics, the following factors were evaluated according to pathological type: number, size and maximum diameter of brain metastases; size and maximum diameter of peritumoral edema; ratio of tumor and peritumoral edema; and asymptomatic ratio. The asymptomatic ratio refers to the proportion of symptom-free patients among the total number of patients with brain metastases. Tumor size was measured as A × B, where A is the maximum diameter of the tumor (mm), recognized as an enhanced lesion on the axial T1-weighted gadolinium-diethylenetriaminepentaacetic acid contrast-enhanced MRI and B is the maximum diameter (mm) perpendicular to A. Edema size was also measured in the same or a different plane across the tumor by using fluid-attenuated inversion recovery (FLAIR) imaging. The ratio of tumor and peritumoral edema (PE-index) was calculated using the equation:

$$\begin{array}{l} \mathrm{PE}–\mathrm{index}=\left\{\sum_{i=1}^{n}\left(\frac{\mathrm{edema}\;\mathrm{size}\;i}{\mathrm{tumor}\;i}\right)\right\}/n\\ \;\;\;\;\;\;\;\;\;\;\;\;\;\;\;\;\;\;\;\;\;\;n=\left(\mathrm{tumor}\;\mathrm{number}\;\leq 5\right) \end{array}$$

If the number of metastases was >5, objects were limited to a maximum of 5 lesions in descending order from the largest tumor. Using this method, when peritumoral edema was almost absent, the PE-index was ∼1.0.

### Assessment of post-treatment factors

To determine post-treatment clinical characteristics, the following factors were evaluated according to histological type: tumor size reduction rate, complete response (CR) rate, intracranial progression-free survival (PFS) and overall survival (OS). Tumor size reduction rate was calculated by the following equation, using images in which the best response was observed: tumor size reduction rate = (sum of pretreatment MRI tumor size- sum of post-treatment MRI tumor size)/(sum of pretreatment MRI tumor size) × 100.

The interval of follow-up MRI was 1–3 months and CR was defined as the status for which the sum of the tumor size was <10 mm^2^. To assess the effect of EGFR-TKIs on survival, a comparison of intracranial PFS and OS between adenocarcinoma patients treated with and those not treated with EGFR-TKIs following WBRT was also performed. Gefitinib (Iressa; AstraZeneca, Macclesfield, UK) was used as an EGFR-TKI in 10 patients and erlotinib (Tarceva; OSI Pharmaceutics Inc., Melville, NY, USA) was used in 7 patients. A combination of gefitinib and erlotinib was used in 2 patients, at a daily dose of 250 and 150 mg, respectively. Intracranial PFS was measured from the initiation of radiotherapy to the date of progression of brain lesions on MRI or death from any cause. OS was measured from the start of radiotherapy to the date of death. Surviving patients were censored at the time of their last follow-up.

### Statistical analysis

Statistical analyses were performed using the software package IBM SPSS Statistics 19. Proportions were compared using the Pearson’s Chi-square test. Survival rates were calculated using the Kaplan-Meier method and compared using the log-rank test. The Kruskal-Wallis test was used for comparison among three categories for continuous variables. When significant differences were detected, the Mann-Whitney U test was used to detect the difference between two categories.

## Results

### Patient characteristics and survival

Patient characteristics are provided in Table I. Adenocarcinoma was the most common pathological type, followed by SCLC and squamous cell carcinoma. Eight patients (11.4%) were classified as recursive partitioning analysis (RPA) class I, 45 (64.3%) were RPA class II and 17 (24.3%) were RPA class III. Motor weakness was the most frequent symptom and brain metastases in asymptomatic patients were detected during the routine follow-up MRI. All patients were Japanese.

The median dose of WBRT was 30 Gy (range, 24–50 Gy). Dose fraction sizes were 2 Gy in 23 patients, 2.4 Gy in 3 patients, 2.5 Gy in 7 patients and 3 Gy in 37 patients. Five patients who presented with large metastases received an additional boost of irradiation limited to large lesions at a dose of 20 Gy in 10 fractions, 9 Gy in 3 fractions or 15 Gy in 5 fractions, depending on the preference of the physicians. Contrast-enhanced MRI scans following WBRT were available for evaluation for 50 patients who did not experience clinical deterioration. The median survival time (MST) for all patients was 26.2 weeks (95% CI, 19–35 weeks), with a median follow-up time of 24 weeks. At the time of assessment, 7 of the 18 censored patients were alive.

Reirradiation for intracranial regrowth following WBRT was performed in 5 patients with adenocarcinoma (4 were treated with a cyberknife and 1 with a γ-knife) and in 4 patients with SCLC (3 were treated with WBRT and 1 with a γ-knife). In adenocarcinoma patients, the MST following the completion of reirradiation was 27 weeks (range, 4–34 weeks) and in SCLC patients, the MST was 21 weeks (range, 8–114 weeks). No significant difference was observed between the two irradiated groups (P=0.711).

### Clinical characteristics according to histological lesion type

Table II shows the clinical characteristics according to histological type. Total tumor size refers to the sum of each tumor size up to 5 lesions and total edema size refers to the sum of each edema size up to 5 lesions. The distribution of the number of brain metastases, tumor size and maximum tumor diameter did not significantly correlate with histological type. However, significant differences in edema size, maximum diameter of edema, PE-index and tumor size reduction rate were observed among the three histological categories (P=0.041, 0.025, <0.001 and <0.001, respectively).

**Figure 1.**
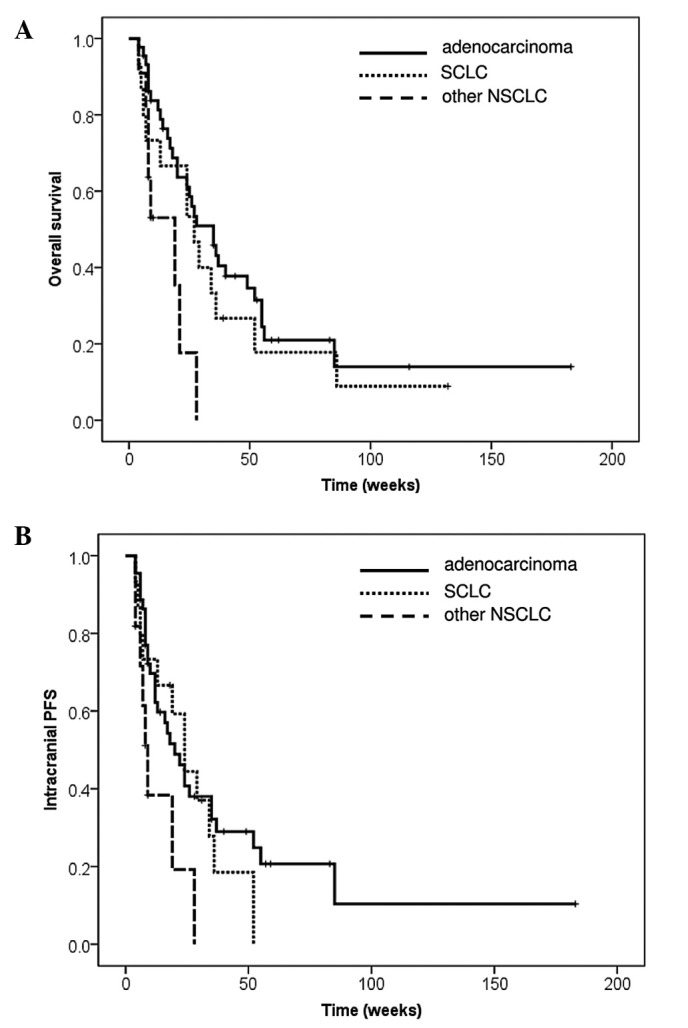
Kaplan-Meier survival curves showing (A) overall survival (OS) and (B) intracranial progression-free survival (PFS) according to histological type. Significant differences were observed between adenocarcinoma and other non-small-cell carcinoma (NSCLC) in intracranial PFS and OS (P= 0.018 and 0.004, respectively). A significant difference was observed in intracranial PFS between small-cell lung carcinoma (SCLC) and other NSCLC (P=0.048).

Steroids were administered to improve symptoms caused by the metastases or the adverse effects of radiotherapy following evaluation of the lesions on MRI in 38 patients. The daily dose of dexamethasone was 2 mg in 14 patients (9 with adenocarcinoma, 2 with SCLC and 3 with other NSCLC), 4 mg in 17 patients (10 with adenocarcinoma, 3 with SCLC and 4 with other NSCLC), 6 mg in 2 patients with adenocarcinoma and 8 mg in 5 patients (3 with adenocarcinoma and 2 with SCLC). No significant differences were observed between these groups using the Chi-square test (P=0.797). Comparison of histological categories revealed differences in clinical characteristics based on histological type. Patients with SCLC exhibited a significantly smaller edema size and maximum edema diameter compared with patients with other NSCLC (P=0.016 and 0.010, respectively), but not compared to those with adenocarcinoma (P=0.065 and 0.134, respectively). By contrast, the tumor size reduction rate was significantly higher in patients with SCLC, compared to those with adenocarcinoma or other NSCLC (P=0.002 and 0.005, respectively). Furthermore, SCLC patients had a significantly lower PE-index compared to those with the other two histological types (P= 0.001). Intracranial PFS and OS were not significantly different between SCLC and adenocarcinoma patients (Fig. 1A and B). However, intracranial PFS and OS differed significantly between adenocarcinoma and other NSCLC patients (P=0.018 and 0.004, respectively). Furthermore, intracranial PFS, unlike OS, was significantly different between SCLC and other NSCLC patients (P=0.048 and 0.071, respectively).

### Effect of EGFR-TKIs on prognosis following WBRT

**Figure 2.**
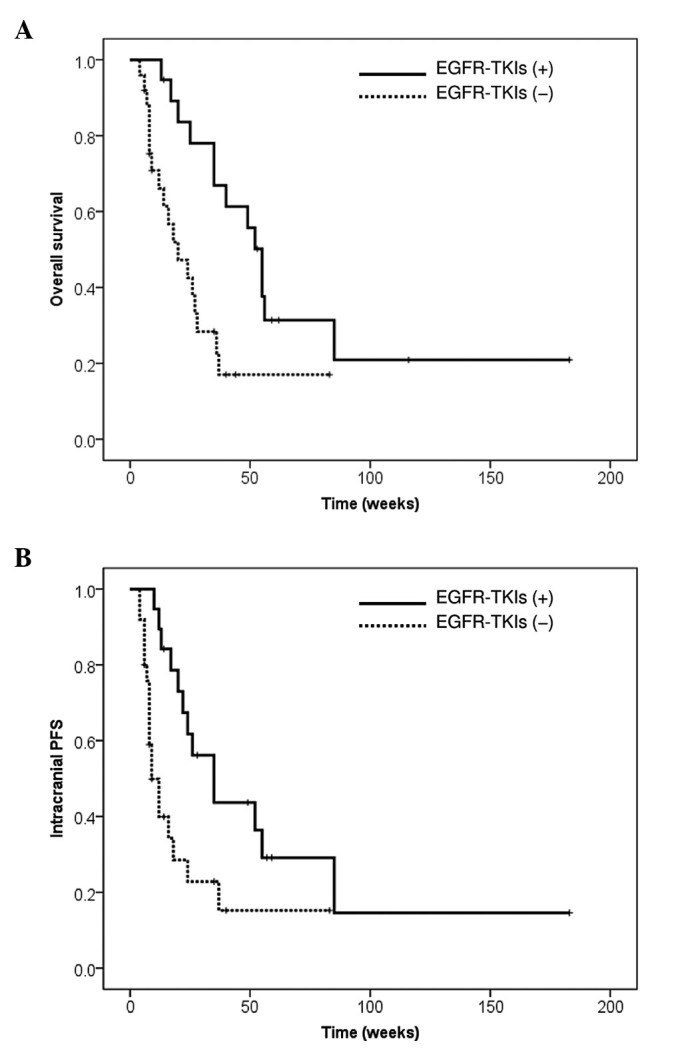
Kaplan-Meier survival curves showing (A) overall survival (OS) and (B) intracranial progression-free survival (PFS), stratified by the administration of epidermal growth factor receptor (EGFR) and tyrosine kinase inhibitors (TKIs), following whole-brain radiotherapy. Significant correlations between OS and the administration of EGFR-TKIs (P=0.004) and between intracranial PFS and the administration of EGFR-TKIs (P=0.008) were observed.

Out of the 44 adenocarcinoma patients, 19 were treated with EGFR-TKIs following WBRT. In 5 of these patients, EGFR mutations were confirmed (exon 21 point mutation, 1 patient; exon 19 deletion, 4 patients). EGFR-TKIs were administered as a second-line treatment in a further 14 patients who had never smoked, after they became resistant to standard platinum-based doublet chemotherapy. EGFR-TKIs were administered to treat locoregional disease in 6 patients, multiple pulmonary metastases in 7 patients, liver or bone metastases with/without locoregional disease in 3 patients and brain metastases in 3 patients. Distributions of the number of brain metastases and the RPA class were not significantly different between EGFR-TKI-treated and non-treated patients (Table III). According to the Kaplan-Meier survival curve, intracranial PFS and OS were significantly higher in EGFR-TKI-treated patients (P=0.008 and 0.004, respectively; Fig. 2A and B). MST and progression-free median survival time were 18.8 (95% CI, 9–28) and 8.9 (95% CI, 7–18) weeks, respectively, in the non-treated group and 52.0 (95% CI, 25–85) and 30.4 (95% CI, 20–85) weeks, respectively, in the EGFR-TKI-treated group.

## Discussion

Although brain metastases from one primary tumor type may behave differently from those derived from another tumor type, the prognosis of patients with brain metastases is generally poor. Although the survival prognosis for lung cancer patients has improved due to the advances in systemic therapy, various cancers behave differently in terms of patterns of systemic disease and response to systemic therapy. These observations suggest that histological type may affect survival following WBRT due to different responses to recent systemic therapies (7–9). However, in a previous review by Gaspar *et al* it was reported that evidence supporting the effect of histopathology on WBRT treatment outcome was insufficient (17). Furthermore, with regards to survival benefit, the role of systemic treatment is controversial (15,18) and WBRT remains the standard palliative treatment.

In this study, we investigated the survival of patients with lung cancer as defined by histological type following WBRT. Bergqvist *et al* previously reported a CR rate of 22% in SCLC, 5% in adenocarcinoma and 0% in squamous cell carcinoma patients (10) based on autopsy results, which is in accordance with the data presented in our study, although our value of the CR rate is slightly higher, due to imaging evaluation. The significant difference in survival following WBRT between adenocarcinoma and other NSCLC patients observed in the present study is in contrast to the results of a previous study by Kepka *et al* which demonstrated no significant difference in survival following WBRT between NSCLC and SCLC, or adenocarcinoma and other NSCLC (11). Differences in the results between these two studies may be due to our use of EGFR-TKIs, which were not routinely used at the time of the study by Kepka *et al* (11). Results of the present study indicated that treatment with EGFR-TKIs leads to an improved prognosis and OS rate in all adenocarcinoma patients. In agreement with our findings, EGFR-TKIs have exhibited increased efficacy when used after WBRT. In addition, several studies reported the favorable effect of EGFR-TKIs on brain metastases (15,19–22) and the improvement in the treatment response to EGFR-TKIs following radiotherapy (23–25).

Preclinical studies have also demonstrated the enhancing effect of previous irradiation on subsequent administration of EGFR-TKIs (26). Ceresoli *et al* conducted a prospective study to evaluate the efficacy of gefitinib in treating brain metastases (27). In their study on 41 patients treated with gefitinib, 18 patients who had previously been treated with WBRT exhibited improved disease control, compared to those who had not undergone WBRT (56 vs. 9%). Ceresoli *et al* hypothesized that WBRT may increase the permeability of the blood-brain barrier, permitting increased drug entry, or that the development of radiation-resistant clones induced by WBRT may be more sensitive to an alternative therapy. Another preclinical study demonstrated that irradiation activates the EGFR by releasing transforming growth factor α (28), whereas the effect of EGFR-TKIs on lung cancer was proven to be dependent on EGFR mutations (29,30).

Differences in the effect of EGFR-TKIs on survival have been observed, depending on whether they were used as first- or second-line treatment and on the presence or absence of EGFR mutations. A previous study suggested that EGFR mutations should be evaluated to determine eligibility when EGFR-TKIs are used as first-line treatment (31). By contrast, other studies demonstrated that EGFR mutations are only associated with response and do not affect survival (9,32). As a second-line treatment, EGFR-TKIs have been shown to have survival benefits. Since EGFR-TKIs were administered as a second-line treatment to several patients in the present retrospective study, EGFR mutations were not adequately assessed.

Furthermore, the results of the Southwest Oncology Group (SWOG) S0023 study, evaluating the efficacy of maintenance gefitinib following chemoradiation therapy, demonstrated inferior survival in patients receiving gefitinib as maintenance therapy (33). This inconsistency may be attributed to differences in patient characteristics, including race. However, the interaction between radiation and subsequent administration of EGFR-TKIs has not been fully elucidated (34,35). Additional subgroup analysis considering these factors may provide useful insight on the subject.

WBRT aims to improve neurocognitive function and it has been reported that tumor size, peritumoral edema and tumor size reduction rate are related to neurocognitive function (36,37). However, few studies have addressed this issue. In our study, pretreatment clinical characteristics were evaluated according to histological type. Peritumoral edema and asymptomatic ratio were less significant in patients with SCLC compared to those in patients with other histological tumor types. Peritumoral edema correlates strongly with neurological morbidity. Tabaka *et al* demonstrated, using specimens resected from patients who underwent surgery, that squamous cell carcinoma infiltrated into the surrounding brain tissue the most aggressively and that the border between brain metastasis and surrounding tissue was well defined in patients with SCLC (38). This histopathological feature may explain our finding of less extensive peritumoral edema in SCLC patients. Moreover, Wegner *et al* also reported a high asymptomatic ratio of up to 73% in a study on stereotactic radiosurgery for patients with brain metastases from SCLC (39).

Taken together, these findings indicate that early detection of brain metastases in patients with SCLC may require regular brain imaging examinations, regardless of the presence or absence of symptoms. Early detection of intracranial recurrence by regular imaging examination and subsequent reirradiation may become one of the strategies in following up patients with SCLC. Due to the poor response to radiotherapy and the limited number of metastases, more aggressive approaches, such as surgery or stereotactic radiosurgery, may be suitable for patients with other NSCLC in the case of recurrence. However, our results must be interpreted with caution, considering the selection bias due to the retrospective nature of this study, race differences and our relatively limited sample size. Thus, additional investigations are required to validate the benefits of such treatment strategies.

In conclusion, in this study we presented the clinical characteristics associated with different histological types of lung cancer, which may provide useful information for the management of brain metastases from lung cancer. Patients with SCLC exhibited a lower degree of peritumoral edema, whereas patients with adenocarcinoma, particularly those treated with EGFR-TKIs, exhibited improved survival rates.

## Figures and Tables

**Table I.** Patient characteristics.

Characteristics	No. of patients	%
Age (years)		
Median, 64 (range, 31–82)	70	100
Gender		
Male	52	74.3
Female	18	25.7
KPS		
100-80	40	57.1
70-50	27	38.6
40-10	3	4.3
Histology		
Adenocarcinoma	44	62.9
Small-cell lung carcinoma	15	21.4
Others	11	15.7
Squamous cell carcinoma	6	
LCNEC	2	
Mucoepidermoid carcinoma	1	
Pleomorphic carcinoma	1	
Large-cell carcinoma	1	
RPA class		
I	8	11.4
II	45	64.3
III	17	24.3
Symptom		
Motor weakness	8	11.4
Headache	6	8.6
Speech difficulties	6	8.6
Light-headedness	6	8.6
Visual disturbance	3	4.3
Vomiting	3	4.3
Numbness	2	2.9
Dizziness and vertigo	2	2.9
Disorientation	2	2.9
Seizure	1	1.4
None	31	44.3

KPS, Karnofsky performance status; LCNEC, large-cell neuroendocrine carcinoma; RPA, recursive partitioning analysis.

**Table II.** Clinical characteristics according to histological type.

Characteristics	Histological type			P-value
	Adenocarcinoma	SCLC	Other NSCLC	
Number of lesions				0.294
1–3	15 (34%)	7 (46%)	7 (64%)	
4–6	9 (20%)	4 (27%)	2 (18%)	
7–10	10 (23%)	0 (0%)	1 (9%)	
≥11	10 (23%)	4 (27%)	1 (9%)	
Total tumor size (mm^2^)				0.650
Median (range)	486 (13–3635)	355 (40–1881)	549 (30–3348)	
Total edema size (mm^2^)				0.041
Median (range)	1795 (13–9894)	593 (40–4352)	4598 (48–10744)	
Tumor max diameter (mm)				0.424
Median (range)	19 (2–53)	20 (6–37)	25 (6–62)	
Edema max diameter (mm)				0.025
Median (range)	35 (3–92)	21 (6–82)	56 (8–95)	
PE-index				<0.001
Median (range)	3.07 (1.00–20.33)	1.30 (1.00–9.80)	5.29 (1.49–19.47)	
Asymptomatic ratio	45%	60%	18%	
RPA class				0.295
I	5 (11%)	3 (20%)	0 (0%)	
II	29 (66%)	10 (67%)	6 (55%)	
III	10 (23%)	2 (13%)	5 (45%)	
Tumor size reduction rate (n=50)				<0.001
Median	22.5%	100%	33.0%	
Mean	22.7%	88.9%	35.8%	
Range	−121 to 100%	52 to 100%	−19 to 95%	
CR rate	5.8%	63.6%	0%	
MST (weeks)	29.2	25.5	10.7	

PE-index, ratio of tumor and peritumoral edema. SCLC, small-cell carcinoma; NSCLC, non-small-cell carcinoma; RPA, recursive partitioning analysis; CR, complete response; MST, median survival time.

**Table III.** Distribution of number of lesions and recursive partitioning analysis class (RPA).

Variables	Without EGFR-TKIs	With EGFR-TKIs	P-value
No. of bone metastases			0.270
1–3	10	5	
4–6	6	3	
7–10	6	4	
≥11	3	7	
RPA class			0.596
I	3	2	
II	15	14	
III	7	3	

EGFR, epidermal growth factor receptor; TKIs, tyrosine kinase inhibitors.
